# Efficacy of Palatal Applications on Fracture Resistance of Reattached Maxillary Central Incisors: An *In Vitro* Study

**DOI:** 10.1155/2017/9124545

**Published:** 2017-12-26

**Authors:** İhsan Yikilgan, Nagihan Guven, Cemile Kedıcı Alp, Emre Tokar, Ozgur Topuz, Hanife Kamak

**Affiliations:** ^1^Department of Restorative Dentistry, Faculty of Dentistry, Gazi University, Ankara, Turkey; ^2^Department of Endodontics, Faculty of Dentistry, Baskent University, Ankara, Turkey; ^3^Department of Prosthodontics, Faculty of Dentistry, Gazi University, Ankara, Turkey; ^4^Department of Endodontics, Faculty of Dentistry, Gazi University, Ankara, Turkey

## Abstract

The purpose of this study was to evaluate the effects of different palatal applications on fracture strength of the fractured anterior tooth. Sixty caries-free human maxillary incisors were used. Endodontic treatments of the teeth were performed. Then, the teeth were divided randomly into five groups (*n* = 12). Crowns of all teeth in groups A–D were cut with diamond discs at a fixed distance of 3 mm from the incisal margin in a plane normal to the buccal surface. In all groups, coronal fragments were reattached to the remaining teeth by bonding with hybrid composite resin. After then, the teeth were restored to the following; group A, bonding and palatal laminate; group B, bonding and creation of a vertical groove; group C, bonding and creation of two slot grooves; group D, bonding only; and group E, intact tooth. It was lesser in group B than in groups C and E (*p* = 0.007 and *p* = 0.006, resp.) and lesser in group D than in groups A, C, and E (*p* = 0.002, *p* < 0.001, and *p* < 0.001, resp.). Within the limitations of this in vitro study, it can be concluded that methods employing palatinal laminate and small grooves are clinically feasible for the reattachment of tooth fragments to incisors.

## 1. Introduction

Tooth fractures are caused by many events, such as accidents, falls, and sports activity (especially martial arts). Many fractures occur in the anterior teeth, especially the maxillary central incisors, because of their anterior and protrusive positions in the dental arch [1, 2]. Trauma may cause small fractures, complicated crown fractures, and/or avulsion [3, 4]. Treatment options are evaluated according to the extent of destruction of the teeth and surrounding tissues. Uncomplicated crown fractures can be repaired with simple restorative treatments, but complicated crown fractures may require multidisciplinary approaches with consideration of periodontal, endodontic, restorative, and/or prosthetic elements [5].

In clinical practice in dentistry, many restorative techniques have been employed in the treatment of coronal fractures. The most frequently applied treatments involve the use of porcelain crowns and composite restorations and the reattachment of tooth fragments. Prosthetic restorations are expensive and nonconservative, and multiple sessions are required for treatment involving such restorations. Composite restorations can be affected by discoloration and aesthetic and adhesive failure. Clinicians often prefer to reattach tooth fragments due to the advantages of this approach [6–8], including conservation of the natural form and optical properties of the tooth, the low cost, and the conservative and practical nature of such restoration [9].

Chosack and Eidelman [10] introduced the method of tooth fragment reattachment in a 1964 case report. Since that time, numerous clinicians have tried different methods, seeking one that offers the best adhesion and greatest strength. Tennery [11] proposed the use of acid etching, and Simonsen [12] additionally proposed the use of circumferential chamfering. Tooth fragments have been reattached using dual-cured composite resin cement [13] and a bonding agent and flowable composite [14]. The use of metal and fiber posts for this purpose has also yielded clinical success [5, 15, 16].

Many *in vitro* studies have been performed with the ultimate aim of increasing the fracture strength of the teeth with reattached fragments. Chazine et al. [17] reported that beveling significantly improved the bond strength of reattached fragments. Stellini et al. [18] reported that the results of overcontouring were more favorable than those of circumferential chamfering in terms of fracture resistance. Loguercio et al. [19] argued that fiber post placement prior to fragment reattachment in endodontically treated teeth was not necessary but that buccal or circumferential chamfering at the fracture line was essential to increase fracture strength. Fennis et al. [20] reported that use of a minifiber-reinforced composite anchor increased the fracture resistance of reattached coronal fragments of incisors. Andreasen et al. [21] reported that laminate veneer application might increase the fracture resistance of reattached coronal incisor fragments.

Preservation of the facial enamel of fractured anterior teeth is important to achieve ideal aesthetics. The aim of this *in vitro* study was to evaluate restoration alternatives, such as the use of palatal laminate and palatal slot cavities, for facial enamel.

## 2. Materials and Methods

The Clinical Research Ethics Committee of Gazi University approved this study (number 25901600/190). Sixty caries-free human maxillary incisors that had been extracted due to periodontal disease or prosthetic treatment were used. The teeth with crown lengths of 10 ± 1 mm, root lengths of 13 ± 1 mm, and no crack, carious lesion, or other structural defect (as observed under ×3.5 magnification) were selected. The teeth were disinfected in 0.5% chloramine for 15 days after extraction and stored in 0.9% saline solution at room temperature for less than 6 months.

Endodontic access cavities were prepared on the specimens, and the working length was established 0.5 mm short of the apical foramen using a visual method. Root canals were instrumented using the crown-down technique with a ProTaper Universal nickel-titanium rotary set (Dentsply, Maillefer). After each file use, the root canals were irrigated with 10 mL 2.5% sodium hypochlorite. An F4 master apical file was used on all specimens. ProTaper F4 gutta percha points (Dentsply, Maillefer) and AH Plus sealer (Dentsply DeTrey) were used for root canal filling, with adaptation to the root canal system 1 mm above the apex. Excess gutta percha at the canal orifice was removed using a heated plugger 1 mm apical to the orifice, and the access was filled with light-cured glass ionomer cement (Ketac Nano; 3M ESPE) and hybrid composite resin (Filtek Z250; 3M ESPE).

After root canal treatment, plastic tubes (40 mm length, 25 mm diameter) were filled with autopolymerizing acrylic resin (Meliodent; Heraeus Kulzer). The teeth were embedded in the plastic tubes up to 2 mm below the cemento-enamel junction (CEJ) with their long axes parallel to the axes of the tubes. Then, the teeth were divided randomly into five groups (*n* = 12 each): group A, bonding and palatal laminate; group B, bonding and creation of a vertical groove; group C, bonding and creation of two slot grooves; group D, bonding only; and group E, intact tooth.

The teeth in group E served as controls, with no treatment application. Crowns of all teeth in groups A–D were cut at a fixed distance of 3 mm from the incisal margin in a plane normal to the buccal surface. Incisions were made under a continuous jet of water using cutting 0.2 mm thick diamond discs.

In all groups, coronal fragments were reattached to the remaining teeth by bonding (Single Bond Universal; 3M ESPE) with hybrid composite resin material (Filtek Z250; 3M ESPE). The fracture surfaces of each coronal fragment and remaining tooth were etched with 35% orthophosphoric acid (3M ESPE). Enamel surfaces were etched for 30 s, and dentin surfaces were etched 15 s. The surfaces were rinsed thoroughly, air-dried gently, and bonded (Single Bond Universal; 3M ESPE). The adhesive system was applied to the tooth according to the manufacturer's instructions and light cured for 20 s.

Hybrid composite resin (Filtek Supreme; 3M ESPE) was applied at the junction of each pair of fragments, and the palatal and labial surfaces were light cured for 30 s. The Curing Light 2500 (3M ESPE) was used for polymerization.

**Figure 1:**
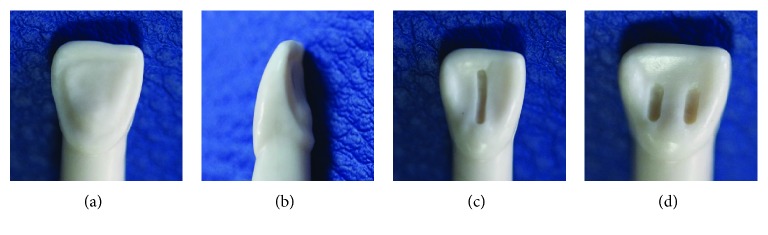
Images of restoration groups: (a, b) palatal laminate, (c) vertical groove, and (d) small grooves.

In group A, the palatal surfaces of teeth were prepared using a 0.5 mm self-limiting depth-cutting bur to define the incision depth and a chamfer diamond bur to refine the preparation for palatal laminate restoration. A new bur was used for each preparation. Finish lines were located to at the proximal contact. The cervical finish lines were located 1 mm above the CEJ. Then, the prepared palatal surfaces were etched for 30 s, rinsed, and dried; one layer of adhesive was applied and polymerized for 20 s; and restoration was completed by polymerizing the hybrid resin for 30 s (Figures 1(a) and 1(b)).

In group B, a vertical groove (1 mm deep, 1 mm wide, and 8 mm long) was made parallel to the long axis of each tooth on the palatal surface using a fissure diamond bur under water cooling. Then, the groove was restored, respectively, etched for 30 s, rinsed, and dried; one layer of adhesive was applied and polymerized for 20 s; and restoration was completed by polymerizing the hybrid resin for 30 s (Figure 1(c)).

In group C, two small grooves (2 mm deep, 2 mm wide, and 5 mm long) were made on both palatal surfaces of each tooth. Then, the cavities were restored, respectively; enamel and dentin were etched for 15 s and 30 s, respectively, then rinsed, and dried; one layer of adhesive was applied and polymerized for 20 s; and restoration was completed by polymerizing the hybrid resin for 30 s (Figure 1(d)).

In group D, only the bonding procedure was performed. On the side to mimic their original form, restorations were completed, and 100% for 72 h in a humid environment was grounded.

The fracture strength of the samples was assessed with a universal testing device (Instron Model 1445; Zwick USA, Atlanta, GA, USA). The long axis of the tooth with the force angle of 45° at a speed of 2.5 mm/min was applied at palatal. The force applied at the time of fracture was recorded in Newton.

### 2.1. Statistical Analysis

The normality of continuous variable distributions was assessed using the Kolmogorov–Smirnov test. The Levene test was used to evaluate the homogeneity of variance. Descriptive statistics were calculated, and values are expressed as means ± standard deviations. Differences in fracture strength among groups were evaluated using the Kruskal–Wallis test; when significant *p* values were obtained, Conover's multiple comparison test was used to determine which group(s) differed from others. Data analysis was performed using IBM SPSS Statistics software (version 17.0; IBM Corporation, Armonk, NY, USA). *p* values < 0.05 were considered to be statistically significant.

## 3. Results and Discussion

### 3.1. Fracture Strength of Experimental Groups

Fracture strengths and fracture types are showed in Table 1. Fracture strength differed among groups (*p* < 0.001). It was lesser in group B than in groups C and E (*p* = 0.007 and *p* = 0.006, resp.) and lesser in group D than in groups A, C, and E (*p* = 0.002, *p* < 0.001, and *p* < 0.001, resp.). The fracture strength of group A was similar to those of groups B, C, and E (*p* = 0.093, *p* = 0.288, and *p* = 0.246). No significant difference was detected between groups B and D or between groups C and E (*p* = 0.119 and *p* = 0.922).

### 3.2. Why We Did This Research

The restoration of fractured anterior teeth is a difficult clinical process. It can be achieved using a ceramic crown, composite resin restoration, or reattachment of the fractured portion. Reattachment enables preservation of the enamel's original shape, color, brightness, and surface texture, as well as long-term maintenance of aesthetic properties. In addition, reattachment has a lower cost and requires less chair time than other restoration alternatives do. Thus, this study was performed to evaluate restoration alternatives involving the reattachment of tooth fragments in complicated crown fractures.

The reattachment of a tooth fragment after trauma is a commonly used restoration approach, but doubts persist about the fracture strength and longevity of various reattachment techniques. Researchers have evaluated these properties in teeth restored with buccal and circumferential chamfers, overcontouring, minifiber anchors, laminate veneer, fiber posts, and internal dentinal grooves [17–21]. In this study, palatal applications were evaluated because preservation of the facial enamel is important for long-term aesthetic success.

### 3.3. Comparison of Restoration Alternatives

In this study, fracture strength was significantly greater in groups A, C, and E than in group D. Vertical grooving in addition to bonding had no significant effect. In terms of fracture strength and fracture type in the experimental groups, the best results were observed in group C. No significant difference was observed between groups A and C, but fracture strength in group C was closest to that in intact teeth (group E) and more catastrophic failures occurred in group A than in group C.

Small grooves substantially increase the fracture resistance of reattached fragments, as they aid interlocking of fractured parts, thereby increasing the resistance to shear forces. Fennis et al. [20] reported that the use of minifiber anchors increased the fracture resistance of incisors with reattached coronal fragments; in this study, the creation of small grooves had the same reinforcing effect. Moreover, Fennis et al. [20] reported difficulty in standardizing the preparation of anchor holes, whereas small groove can be created in a standardized manner and also it is so practical.

In this study, fracture strength was greater in the palatal laminate group (group A) than in the bonding only group (group D). In previous studies, fracture strength increased with the bonded surface [19, 21–23]. Andreasen et al. [21] reported that laminate veneer application improved the fracture strength of reattachment fragments. Pusman et al. [22] and Reis et al. [23] reported that overcontouring resulted in greater fracture strength than did simple reattachment. Increasing the bonded surface may reduce stress on the fracture line. In previous studies, additional bonded surfaces were prepared on facial enamel. Researchers have also reported that an increased bulk of composite material on the facial surface may cause future aesthetic problems [23]. Palatal laminate may not cause long-term aesthetic problems because its boundaries are in invisible regions.

### 3.4. Why the Sectioning Method Was Used

In previous studies for the fracture scenario, sectioning and fracturing methods were used [22–25]. Reis et al. [23] defined a methodology for reattachment studies that has been accepted by many researchers. In this method, sound teeth are fractured with a universal testing device and refractured after restoration. Thus, each tooth serves as its own control. In addition, the fractures are similar to those encountered in clinical practice. In addition, the fit between fragments produced with the fracturing method is better than that of fragments produced with the sectioning method [2]. However, the fracturing method has some disadvantages. Some teeth may be excluded from a study because the creation of fractures of the same type and size in all teeth is difficult. For example, Reis et al. [23] reported that 25 of 60 teeth were excluded after the first facture. Ethics committees do not typically approve studies in which a high proportion of specimens may be excluded. In this study, the teeth were sectioned with a diamond saw instead of fractured because the collection of a sufficient number of intact maxillary incisors is difficult and because this approach enabled the establishment of a standard clinical fracture scenario.

### 3.5. Material Selection

In this study, a hybrid resin composite and a bonding agent were used for reattachment. Previous studies have demonstrated that the reattachment technique used for fractured teeth is more important than the type of material used [2, 17, 26]. A hybrid resin composite was used in this study because it was more visible on the vestibular surface of the fracture line; a more stable material (for aesthetic and mechanical) may be a better choice. In addition, acid etching was used because it may increase adhesion to enamel and remove the smear layer formed during sectioning.

## 4. Conclusion

Within the limitations of this *in vitro* study, it can be concluded that methods employing palatal laminate and small grooves are clinically feasible for the reattachment of tooth fragments to incisors, as they increase fracture strength and cause no aesthetic problem.

## Figures and Tables

**Table 1:** Fracture strength measurements and fracture types regarding for groups. *n*: number of cases; data were shown as mean ± SD, and the different uppercase letters indicate statistically significant difference between groups (*p* < 0.05).

	*n*	Mean ± SD	Fracture type	
			Restorable	Unrestorable
Group A	12	562.95 ± 159.53^AC^	5	7
Group B	12	446.61 ± 123.44^BC^	7	5
Group C	12	668.54 ± 240.09^A^	8	4
Group D	12	355.91 ± 114.88^B^	9	3
Group E	12	682.78 ± 239.81^A^	4	8
